# SIK3 and Wnk converge on Fray to regulate glial K^+^ buffering and seizure susceptibility

**DOI:** 10.1371/journal.pgen.1010581

**Published:** 2023-01-10

**Authors:** Lorenzo Lones, Aaron DiAntonio

**Affiliations:** 1 Department of Developmental Biology, Washington University School of Medicine, St. Louis, Missouri, United States of America; 2 Needleman Center for Neurometabolism and Axonal Therapeutics, Washington University School of Medicine, St. Louis, Missouri, United States of America; Harvard Medical School, Howard Hughes Medical Institute, UNITED STATES

## Abstract

Glial cells play a critical role in maintaining homeostatic ion concentration gradients. Salt-inducible kinase 3 (SIK3) regulates a gene expression program that controls K^+^ buffering in glia, and upregulation of this pathway suppresses seizure behavior in the *eag*, *Shaker* hyperexcitability mutant. Here we show that boosting the glial SIK3 K^+^ buffering pathway suppresses seizures in three additional molecularly diverse hyperexcitable mutants, highlighting the therapeutic potential of upregulating glial K^+^ buffering. We then explore additional mechanisms regulating glial K^+^ buffering. Fray, a transcriptional target of the SIK3 K^+^ buffering program, is a kinase that promotes K^+^ uptake by activating the Na^+^/K^+^/Cl^-^ co-transporter, Ncc69. We show that the Wnk kinase phosphorylates Fray in *Drosophila* glia and that this activity is required to promote K^+^ buffering. This identifies Fray as a convergence point between the SIK3-dependent transcriptional program and Wnk-dependent post-translational regulation. Bypassing both regulatory mechanisms via overexpression of a constitutively active Fray in glia is sufficient to robustly suppress seizure behavior in multiple *Drosophila* models of hyperexcitability. Finally, we identify cortex glia as a critical cell type for regulation of seizure susceptibility, as boosting K^+^ buffering via expression of activated Fray exclusively in these cells is sufficient to suppress seizure behavior. These findings highlight Fray as a key convergence point for distinct K^+^ buffering regulatory mechanisms and cortex glia as an important locus for control of neuronal excitability.

## Author summary

K^+^ homeostasis is critical for maintaining healthy levels of neuronal activity. In *Drosophila* glia, SIK3-HDAC4 signaling regulates a gene expression program that controls K^+^ buffering. In *eag Shaker*, a classic model of hyperexcitability, enhancing glia’s capacity to buffer K^+^ is sufficient to suppress seizure behavior. Here we demonstrate that boosting glial K^+^ buffering can suppress seizures in three additional hyperexcitable mutants, highlighting the therapeutic potential of enhancing glial K^+^ buffering. We show *Fray*, a transcriptional target of SIK3-HDAC4 signaling, is a substrate for the kinase Wnk in glia, and that Wnk-dependent regulation of Fray is required for proper K^+^ buffering. We bypass both this transcriptional and post-translation regulation of Fray by expressing a constitutively active transgene of Fray and demonstrate that this is sufficient to suppress seizure behavior in all three mutants. Finally, we identify cortex glia as a key cell type in which boosting K^+^ buffering can suppress seizures. These findings demonstrate that *SIK3* and *Wnk* converge on *Fray* to regulate K^+^ buffering and seizure susceptibility.

## Introduction

Cell volume homeostasis is a challenge faced by all cells. Genetic mutations in genes regulating solute and ion transport cause a variety of human diseases including hypertension, long QT syndrome, and cerebral ischemia [1–3]. In the nervous system, ion transport is central to neuronal function, as it underlies both action potential propagation and evoked neurotransmitter release. In particular, the regulation of K^+^ is important for maintaining healthy, physiological levels of neuronal activity. Following trains of action potentials, extracellular K^+^ levels can increase as neurons release K^+^ into the extracellular space to repolarize the membrane. This efflux of K^+^ creates osmotic pressure that induces the flux of water molecules into the extracellular space. Glial cells express a suite of channels and specialized transporters that play an essential role in removing K^+^ and water from the extracellular space to restore homeostatic ionic and osmotic gradients [1,4]. Defects in glial water and ion homeostasis can lead to devastating consequences such as ionic edema and neuronal hyperexcitability, a cardinal hallmark of seizure disorders [5,6].

Much is known about the molecular mechanisms of ion homeostasis, particularly the identity of the key channels/transporters and their upstream regulators. Among these regulators is With-No-Lysine kinase (WNK), which is well studied in the mammalian kidney as a master regulator of renal ion transport [7]. Wnk phosphorylates the Ste20-related proline/alanine-rich kinase (SPAK) which leads to the phosphorylation and activation of cation-chloride transporters such as NKCC1 [8]. Activation of NKCC1 promotes K^+^ uptake. Wnk-SPAK signaling is evolutionarily conserved as Wnk also signals through Fray (SPAK) in the *Drosophila* Malpighian tubule to regulate transepithelial ion transport [3,9,10]. In the peripheral nervous system, loss of Fray results in nerve swellings due to ionic edema, and this phenotype is suppressed by glial-specific expression of Ncc69, the *Drosophila* orthologue of NKCC1 [11,12]. While the activities of these effectors of ion homeostasis are reasonably well understood, much less is known about the transcriptional regulation of this process.

Recently, we identified a signal transduction cascade in which the AMPK-family member kinase SIK3 and the histone deacetylase HDAC4 regulate the expression of water and K^+^ buffering genes in peripheral *Drosophila* glia [13]. SIK3 promotes K^+^ buffering by sequestering HDAC4 in the cytoplasm, thereby relieving HDAC4 inhibition of the nuclear transcription factor Mef2. Active Mef2 promotes the expression of water and K^+^ buffering genes including the water channel aquaporin and the kinase Fray/SPAK. Loss of SIK3 in glia disrupts K+ buffering and leads to neuronal hyperexcitability and nerve edema, highlighting the key role SIK3 plays in water/ion homeostasis for the nervous system. Interestingly, we demonstrated in the *Drosophila* seizure model *Eag Shaker*, a double mutant for both ERG and Shaker-type K^+^ channels, that the glial SIK3-HDAC4 pathway is inactivated and re-activation of the glial SIK3-HDAC4 pathway suppresses seizure behavior and significantly extends lifespan [14]. The dramatic seizure suppression observed in *Eag Shaker* motivated us to explore whether bolstering the glial capacity to buffer K^+^ could also suppress seizure behavior in molecularly distinct seizure mutants.

Here we demonstrate that two pathways converge on Fray to regulate K^+^ buffering and seizure susceptibility, and that boosting glial K^+^ buffering can suppress seizure behavior in three mechanistically distinct *Drosophila* models of hyperexcitability. We show that activation of the SIK3 transcriptional program in glia is sufficient to suppress seizure behavior in a variety of seizure mutants. We next investigate the function of Wnk in *Drosophila* glia as a candidate post-translational regulator of ion buffering. We demonstrate that Wnk phosphorylates Fray in glia to regulate K^+^ buffering. As we previously demonstrated that SIK3-HDAC4 regulates the transcription of Fray, this finding suggested that Fray may be a critical convergence target of both SIK3 and Wnk. By overexpressing a constitutively active Fray, we bypassed the transcriptional regulator, SIK3, and posttranslational regulator, Wnk, and find that active Fray is sufficient to potently suppress seizure behaviors in multiple hyperexcitable mutants. Finally, we identify cortex glia, which wrap the neuronal cell bodies in the central nervous system [15,16] as an important glial subtype for regulation of seizure behavior via the SIK3 and Fray-dependent pathways. Taken together, this study demonstrates that Fray, a critical downstream target of SIK3 and Wnk, can regulate K^+^ buffering in cortex glia and modulate seizure susceptibility in multiple *Drosophila* models of epilepsy.

## Results

### Hyperexcitability inhibits glial K^+^ buffering

**Fig 1:**
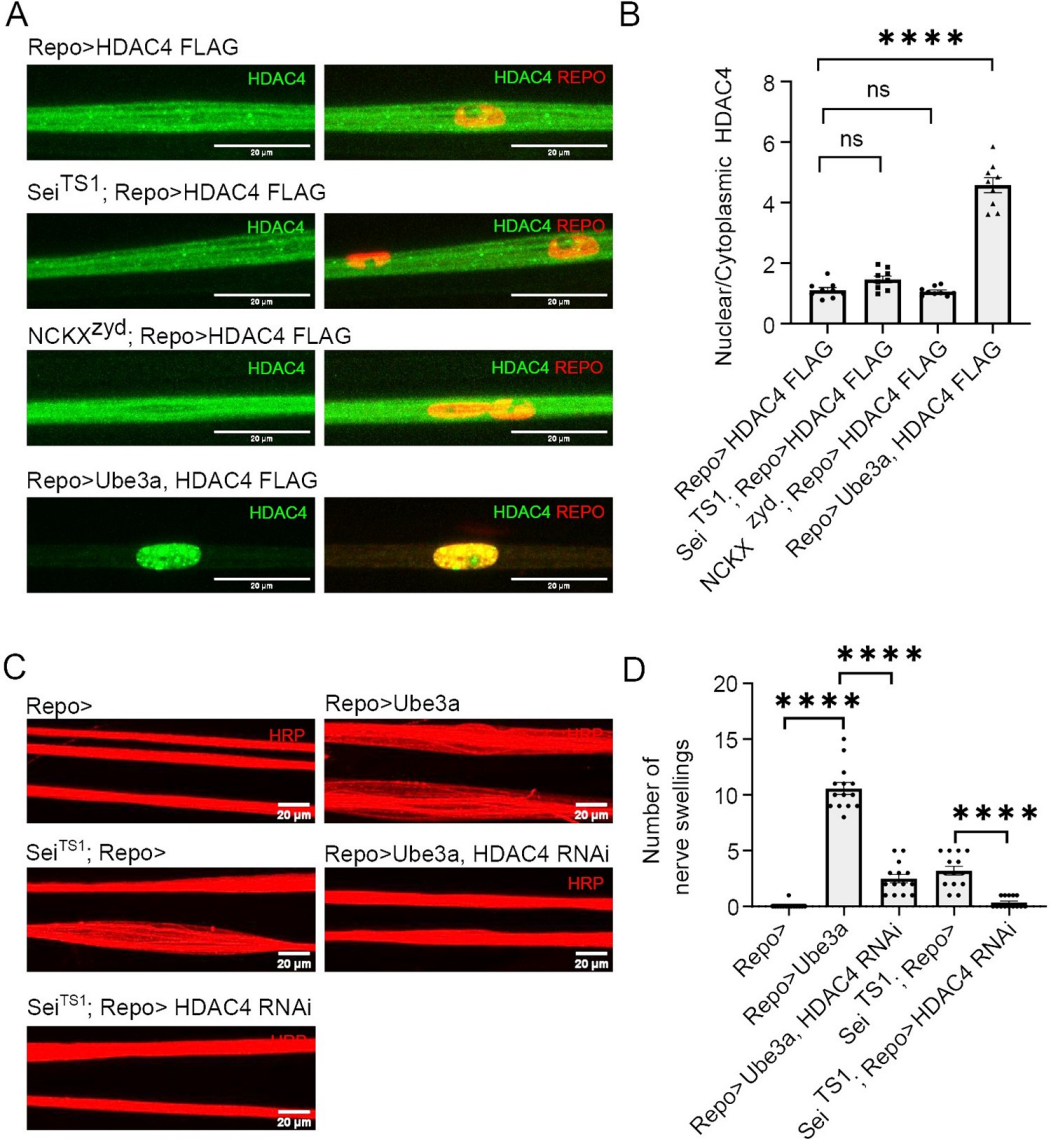
Hyperexcitability influences glial K^+^ buffering program. (A) Representative images of larval peripheral nerves demonstrating the effects of seizure mutants on HDAC4 localization in glia. Pan glial driver, Repo-Gal4, was used to express a FLAG-tagged HDAC4 (green). Glial nuclei are stained with Repo (red). Scale bars 20μm. (B) Quantification of nucleo:cytoplasmic ratio of HDAC4 for genotypes in (A). Data are represented as fold changes relative to *Repo>HDAC4*. n≥6. Each data point represents one larvae in which 4–5 nerves were assessed. Statistical significance was determined by one-way ANOVA with Dunnett’s multiple comparisons test; NS = not significant, ****, p<0.0001. Data are mean± SEM. (C) Representative images of larval peripheral nerves stained for the nerve membrane marker HRP. Repo-Gal4, a pan glial driver, was used to express LexA RNAi (Repo>) or HDAC4 RNAi in both hyperexcitability mutants. (D) Quantification of nerve swellings for genotypes in (C). n≥15. Statistical significance was determined by one-way ANOVA with Šídák’s multiple comparisons; ****, p<0.0001. HDAC4 knockdown suppressed nerve swellings compared to seizure mutants expressing control transgene, UAS-LexA RNAi.

The SIK3/HDAC4 glial K^+^ buffering program is inhibited in *eag shaker*, a double mutant for the ERG and Shaker-type K^+^ channels [14]. Inhibition of this program occurs via shuttling of HDAC4 to nucleus, and can lead to peripheral nerve edema that is observed as nerve swellings. Here we tested whether inhibition of this program is a general feature of hyperexcitable mutants with altered K^+^ homeostasis. We assessed HDAC4 localization and nerve swellings in three seizure mutants defective for K^+^ homeostasis; *NCKX*^*zydeco*^ (zyd) which disrupts a K^+^-dependent Na^+^/Ca^2+^ exchanger [17,18], *Seizure* (Sei^TS1^) which disrupts an H-type voltage-gated K^+^ channel [19,20], and a model of dup15q syndrome in which glial overexpression of the ubiquitin ligase and human disease gene *ube3a* leads to degradation of the Na^+^/K^+^ ATPase pump [21,22]. All three mutants exhibit pronounced seizure phenotypes and are predicted to perturb K^+^ flux. First, we examined HDAC4 localization in peripheral nerve glia expressing a FLAG-tagged HDAC4. In control flies (*Repo*>), HDAC4 is present at similar levels in the cytosol and nucleus. In Ube3a overexpression mutants, HDAC4 is enriched in the nucleus and so has a significantly increased nuclear to cytoplasmic ratio (Fig 1A and 1B). In contrast, there is no difference between the HDAC4 nuclear to cytoplasmic ratio in the Sei^TS1^ or NCKX^zyd^ as compared to control flies, suggesting the SIK3 glial K^+^ buffering program is unaffected by these mutants in these glia. In conjunction with our prior findings that the SIK3 K^+^ buffering program is turned off in *eag shaker* double mutants [14], these data show that neuronal excitability inhibits glial K^+^ buffering in some, but not all, glial cells in some seizure mutants.

We next assessed nerve swellings in these seizure mutants. Nerve morphology is another readout of K^+^ buffering, as inhibition of glial K^+^ buffering leads to ionic edema as the extracellular accumulation of K^+^ draws osmotically obliged water molecules into the extracellular space leading to distinct nerve swellings [13]. While NCKX^zyd^ did not exhibit peripheral nerve swellings, Dup15q and Sei^TS1^ mutants both exhibited nerve swellings that resembled the SIK3 mutant phenotype (Fig 1C), with swellings occurring in discrete locations throughout the peripheral nerve in no obvious pattern. The nuclear localization of HDAC4 in Dup15q suggests that the nerve swellings were the result of an inactive K^+^ buffering program. Thus, we hypothesized that re-activating the K^+^ buffering program would suppress nerve swellings. To activate the K^+^ buffering program in glia, we knocked down HDAC4 with the pan-glial driver Repo-Gal4. When the K^+^ buffering program was activated, we saw a significant reduction in the number of nerve swellings in the dup15q syndrome model (Fig 1D). We were intrigued to observe nerve swellings in the Sei^TS1^ mutant because HDAC4 was unaffected in peripheral glia in this mutant. Hence, the SIK3 K^+^ buffering program was apparently functional in the Sei^TS1^ mutant and so K^+^ homeostasis in the nerve is likely disrupted via a different mechanism. This mutant allowed us to test the hypothesis that enhancing the SIK3 K^+^ buffering program beyond basal levels could suppress nerve swellings. To activate glial K^+^ buffering, we expressed HDAC4 RNAi using the pan glial driver, *Repo-Gal4*. When the K^+^ buffering program was further activated in Sei^TS1^, the number of nerve swellings per larvae was decreased compared to Sei^TS1^mutants expressing a control RNAi (Fig 1D). This suppression, despite the presence of an active SIK3 K^+^ buffering program at baseline, suggests that enhancing the transcriptional K^+^ buffering program may be a broadly useful intervention for suppressing the consequences of hyperexcitability.

### Activating the glial SIK3 K^+^ buffering program suppresses seizure behavior in diverse mutants

**Fig 2:**
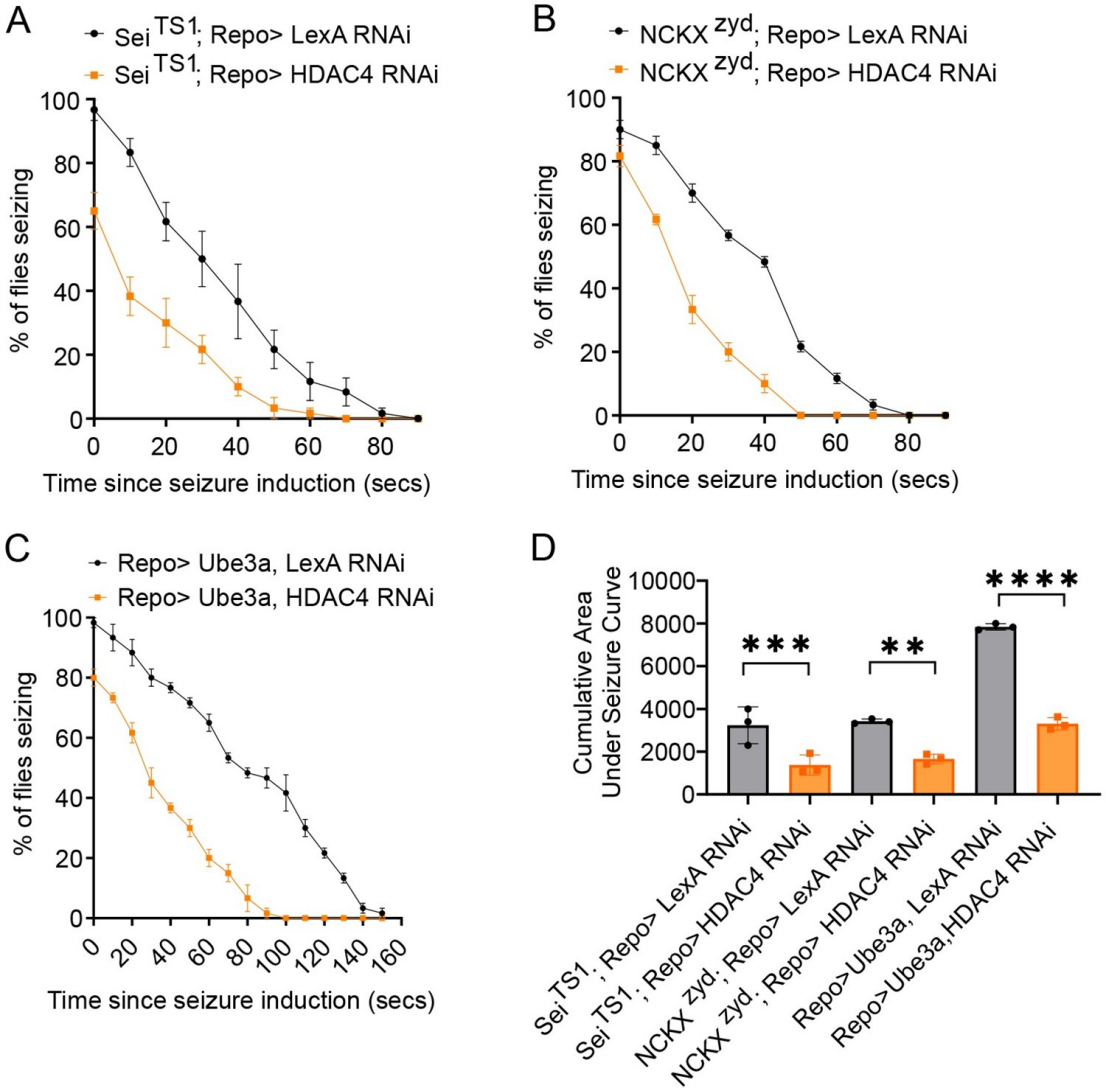
Enhancing glial K^+^ buffering suppresses seizure behavior. (A) Time course of heat shock induced seizure behaviors. Pan glial driver, Repo-Gal4, was used to express control transgene, UAS-LexA RNAi (Sei^TS1^Repo>), or UAS-HDAC4 RNAi (Sei^TS1^Repo>HDAC4 RNAi). After seizure induction, the number of flies seizing were recorded every 10 seconds and plotted as a percentage of seizing flies. n≥ 3 groups of 10–20 flies per genotype per time point. (B) Time course of heat shock induced seizure behaviors. Pan glial driver, Repo-Gal4, was used to express control transgene, UAS-LexA RNAi (NCKX^zyd^Repo>), or UAS-HDAC4 RNAi (NCKX^zyd^;Repo>HDAC4 RNAi). After seizure induction, the number of flies seizing were recorded every 10 seconds and plotted as a percentage of seizing flies. n≥ 3 groups of 10–20 flies per genotype per time point. (C) Time course of vortex-induced seizure behaviors. Pan glial driver, Repo-Gal4, was used to express control transgene, UAS-LexA RNAi (Repo> Ube3A, LexA RNAi), or constitutively active Fray (Repo> Ube3A, Fray^T206E^). After seizure induction, the number of flies seizing were recorded every 10 seconds and plotted as a percentage of seizing flies. n≥ 3 groups of 10–20 flies per genotype per time point. (D) Quantification of area under seizure curve for genotypes in (A-C). Statistical significance was determined by one-way ANOVA with Sidak’s multiple comparisons test; ** p = 0.0010, *** p = 0.006, **** p<0.0001. Data are mean ±SEM.

In *eag shaker* mutants, hyperexcitability inhibits the glial SIK3 K^+^ buffering program. Reactivating the program suppresses seizure behavior and extends lifespan [13]. Moreover, enhancing the SIK3 K^+^ buffering program in the Sei^TS1^ mutant suppressed nerve swellings even though the SIK3 program was not turned off (Fig 1D). This finding led us to hypothesize that activating the program could suppress seizure behavior across a range of distinct seizure mutants. The three mutants we characterized above all show seizures, but otherwise have distinct SIK3 pathway-related phenotypes in the peripheral nerve. In the Dup15q model, the SIK3-HDAC4 glial K^+^ buffering program is *inhibited* and the mutant *exhibits* nerve swellings. In Sei^TS1^ mutants, the SIK3-HDAC4 glial K^+^ buffering program is *active* and yet the mutant still *exhibits* nerve swellings. Finally, in the NCKX^zydeco^ mutant the SIK3-HDAC4 glial K^+^ buffering program is *active* and the mutant *does not exhibit* nerve swellings (Fig 1A–1D). This phenotypic variability a) highlights that there is not an absolute concordance between the presence of nerve swellings and HDAC4 nuclear localization in peripheral glia and b) allowed us to test the hypothesis that activation of the SIK3 K^+^ buffering program would be a broadly useful intervention for inhibiting seizures. To test this hypothesis, we activated the glial SIK3 K^+^ buffering program in each of these seizure mutants by knocking down glial HDAC4 and assayed seizure behavior. In all three mutants, enhancing the glial K^+^ buffering program dramatically suppressed seizure behaviors. To quantify this effect, seizures were induced, and the percentage of flies seizing was recorded every ten seconds (Fig 2A–2C). The cumulative area under the seizure curve was measured and reported as the integrated seizure response. This integrated measure accounts for both the initial fraction of flies that seize as well as the recovery time. In each seizure mutant, the integrated seizure response was significantly decreased when the glial K^+^ buffering program was activated, compared to seizure mutants expressing a control RNAi (Fig 2D). These data suggest that regardless of the status of the SIK3-HDAC4 glial K^+^ buffering pathway in peripheral nerve glia, enhancing the glial capacity to buffer K^+^ is sufficient to suppress seizure behavior. Having demonstrated the broad importance of glial K^+^ buffering in seizure susceptibility, we next investigated other genes implicated in the regulation of glial K^+^ buffering.

### Wnk signals through Fray to regulate glial K^+^ buffering

**Fig 3:**
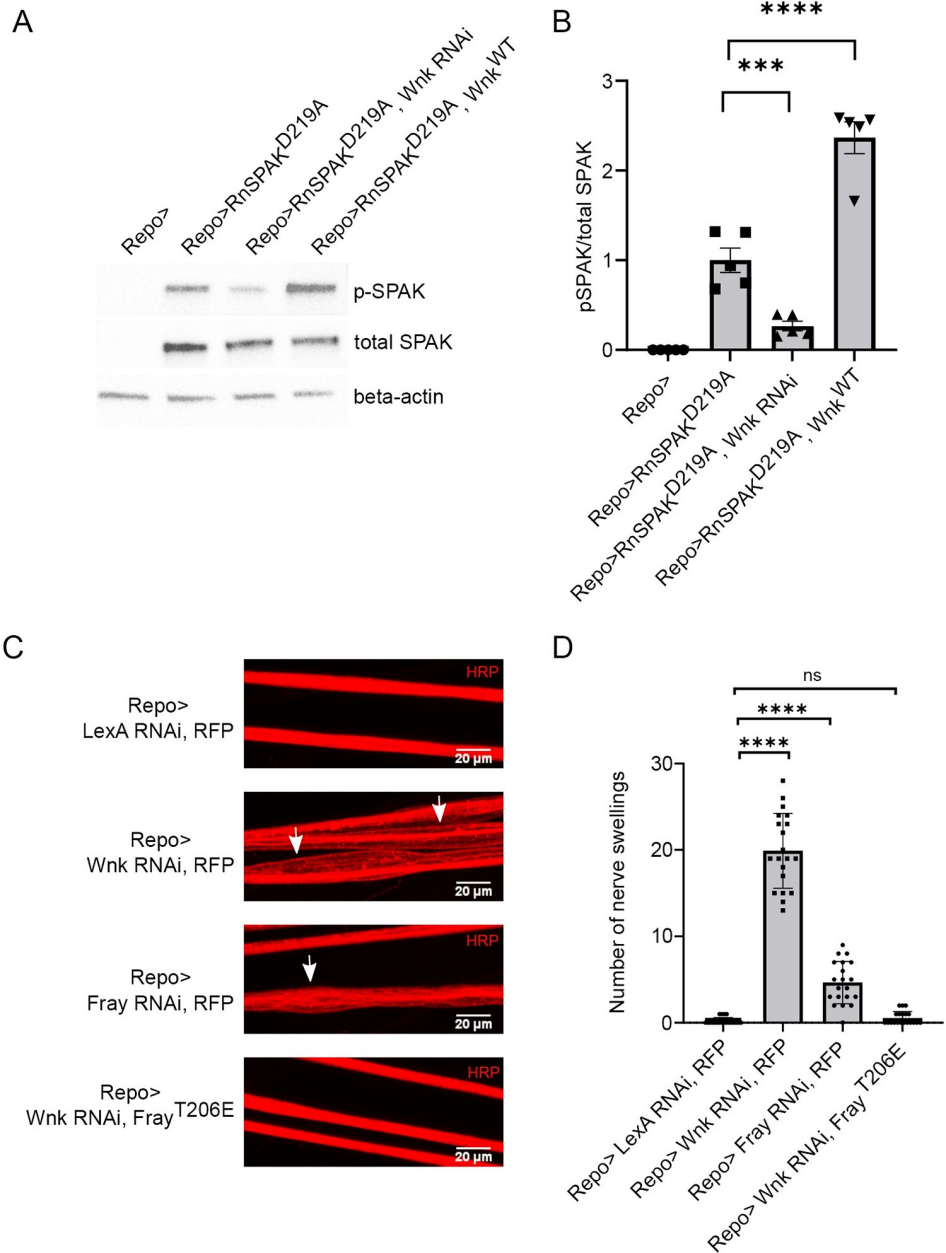
Wnk signals through Fray to regulate glial potassium flux. (A) Representative Western blots stained for phosphorylated rat Ste20-related proline/alanine-rich kinase (p-SPAK), total rat Ste20-related proline/alanine-rich kinase (t-SPAK), and β-actin. The pan glial driver Repo-Gal4 was used to express kinase-dead rat SPAK (RnSPAK^D219A^) as a substrate for endogenous *Drosophila* Wnk. Western blots were first probed for p-SPAK, stripped, and re-probed for t-SPAK. (B) Quantification of the mean (±SEM) ratio of pSPAK/total SPAK for genotypes in (A). Phosphorylation of SPAK decreased with Wnk knockdown (Repo>RnSPAK^D219A^,Wnk RNAi) and increased when Wnk was overexpressed (Repo>RnSPAK^D219A^, Wnk^WT^). Data points represent biological replicates of separate lysate preps. Statistical significance was determined by one-way ANOVA with Sidak’s multiple comparisons test. (C) Representative images of larval peripheral nerves stained for membrane marker HRP. Repo-Gal4, a pan glial driver, was used to express a control transgene, UAS-LexA RNAi (Repo>), UAS-Wnk RNAi (Repo>Wnk RNAi), UAS-Fray RNAi (Repo>Fray RNAi), and co-expression of Wnk RNAi and constitutively active Fray (Repo> Wnk RNAi, Fray^T206E^). Loss of Wnk and Fray in glia causes nerve swellings (arrow). Swellings are suppressed by expressing constitutively active Fray. Scale bars 20μM. (D) Quantification of nerve swellings for genotypes in (C). n≥ 20. Statistical significance was determined by one Way ANOVA with Dunnett’s multiple comparisons; ****, p<0.0001.

Fray (SPAK in mammals), a downstream target of the SIK3-HDAC4-Mef2 pathway, is a master regulator of cation-chloride cotransporters [7] and is required for proper glial K^+^ buffering in *Drosophila* [23]. Wnk directly phosphorylates and activates Fray/SPAK in mammals as well as in the *Drosophila* renal system, but little is known about Wnk function in glia. To assess Wnk activity in glia, we adopted an assay previously developed in the *Drosophila* renal tubule [24] in which a kinase-dead rat SPAK (rSPAK^D219A^) is used as a catalytically-inactive reporter substrate for endogenous *Drosophila* Wnk. We expressed (rSPAK^D219A^) in glia and assessed the ratio of phosphorylated SPAK to total SPAK in the CNS from control flies, as well as flies with glial-specific knockdown or overexpression of Wnk. With knockdown of Wnk there is a significant decrease in the levels of p-SPAK, while upon Wnk overexpression there is a significant increase in the levels of p-SPAK (Fig 3A and 3B). Hence, endogenous Wnk is active in *Drosophila* glia, and so likely phosphorylates Fray, which shares this phosphorylation site with SPAK.

If Wnk functions upstream of Fray in glia to regulate K^+^ buffering, then we predict that losing Wnk in glia will phenocopy loss of Fray and cause peripheral nerve swellings. To test this hypothesis, first we drove a UAS-Fray RNAi transgene in glia from the pan-glia driver Repo Gal-4 and observed nerve swellings, consistent with prior findings [11] (Fig 3C and 3D). Driving the expression of a UAS-Wnk RNAi transgenes with Repo Gal-4 also led to localized nerve swellings (Fig 3C and 3D). A second, non-overlapping UAS-Wnk RNAi transgene also showed a large increase in the number of nerve swellings (BDSC #42521, 11.80+/- 2.5 swellings, n = 19, p < 0.0001). If Wnk signals through Fray in glia, then expression of a constitutively active Fray transgene that does not need to be phosphorylated by Wnk should suppress the Wnk loss-of-function phenotype. Such a Fray transgene exists in which the threonine phosphorylated by Wnk is replaced with a glutamic acid as a phosphomimetic (Fray^T206E^, [24]). Expression of Fray^T206E^ in glia fully suppressed the nerve swelling phenotype due to Wnk knockdown (Fig 3C and 3D). This epistatic analysis in conjunction with the biochemical studies above demonstrate that Fray is an essential downstream target of Wnk in glia for the regulation of K^+^ buffering.

### Activated Fray is sufficient to suppress seizure behavior

**Fig 4:**
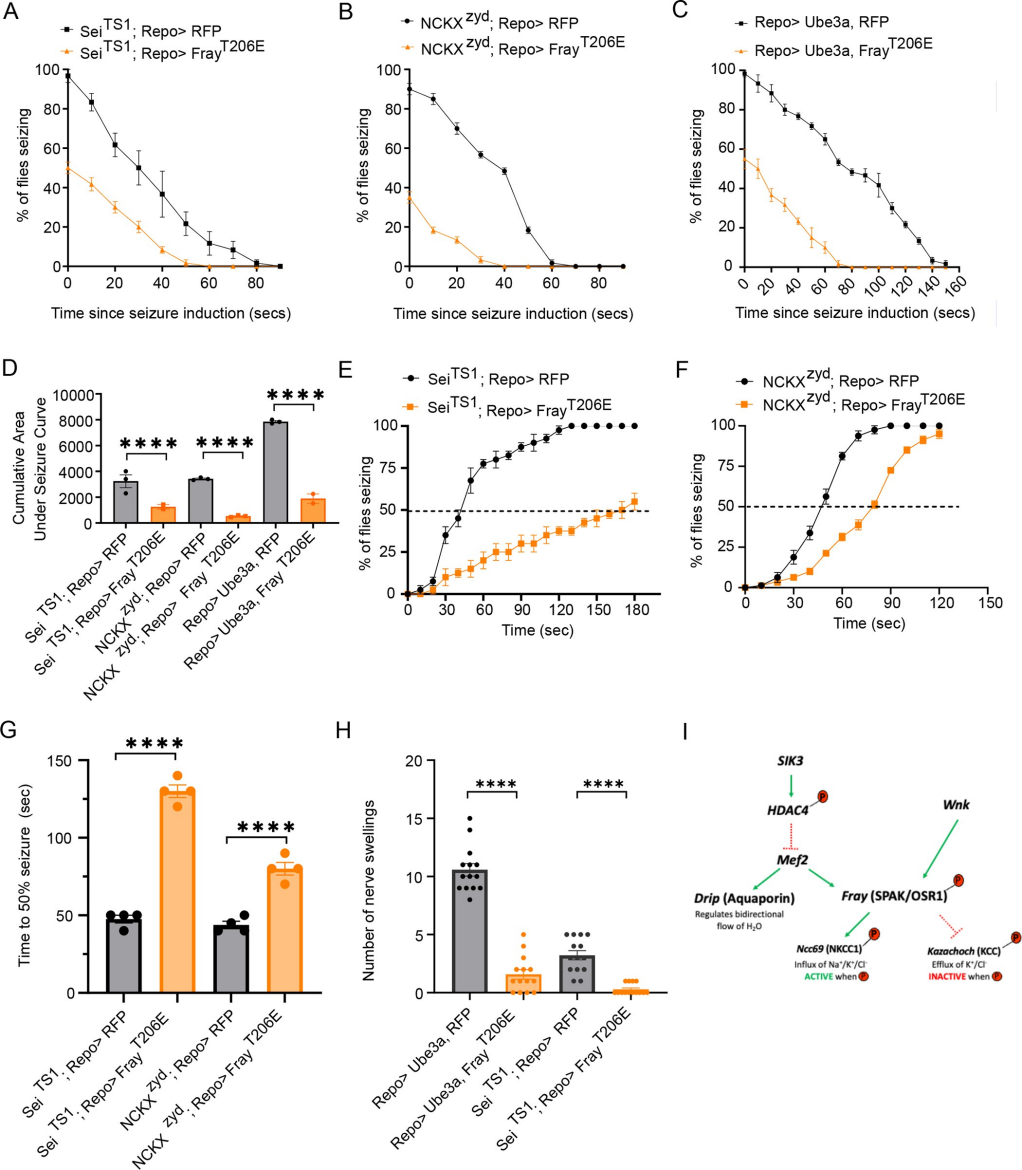
Activating Fray is sufficient to suppress seizure behavior. (A) Time course of heat shock induced seizure behaviors. The pan glial driver Repo-Gal4 was used to express a control transgene, UAS-LexA RNAi (Sei^TS1^;Repo>), or constitutively active Fray (Sei^TS1^;Repo>Fray^T206E^). After seizure induction, the number of flies seizing were recorded every 10 seconds and plotted as a percentage of seizing flies. n≥ 3 groups of 10–20 flies per genotype per time point. (B) Time course of heat shock induced seizure behaviors. The pan glial drive Repo-Gal4 was used to express a control transgene, UAS-LexA RNAi (NCKX^zyd^Repo>), or constitutively active Fray (NCKX^zyd^;Repo>Fray^T206E^). After seizure induction, the number of flies seizing were recorded every 10 seconds and plotted as a percentage of seizing flies. n≥ 3 groups of 10–20 flies per genotype per time point. (C) Time course of vortex-induced seizure behaviors. The pan glial drive Repo-Gal4, was used to express a control transgene, UAS-LexA RNAi (Repo> Ube3A, LexA RNAi), or UAS-Fray^T206E^ (Repo> Ube3A, Fray^T206E^). After seizure induction, the number of flies seizing were recorded every 10 seconds and plotted as a percentage of seizing flies. n≥ 3 groups of 10–20 flies per genotype per time point. (D) Quantification of area under the seizure curve for genotypes in (A-C). Statistical significance was determined by one-way ANOVA with Sidak’s multiple comparisons test; **** p<0.0001. Data are mean ±SEM. (E) Time course of heat-shock induced seizures following overexpression of a control transgene UAS-RFP (Sei^TS1^;Repo>) or constitutively active *Fray* (Sei^TS1^Repo>Fray^T206E^), with dotted line indicating 50% level. Vials of 10–20 flies were submerged in 42°C and the number of seizing flies were recorded every 10 seconds for 3 minutes. (F)Time course of heat-shock induced seizures following overexpression of a control transgene UAS-RFP (NCKX^zyd^; Repo>) or constitutively active *Fray* (NCKX^zyd^; Repo>Fray^T206E^), with dotted line indicating 50% level. Vials of 10–20 flies were submerged in 38°C and the number of seizing flies were recorded every 10 seconds for 3 minutes. (G) Direct comparisons of time for 50% of flies to start seizing. Statistical significance was determined by one-way ANOVA with Sidak’s multiple comparison’s test; **** p<0.0001. (H) Quantification of nerve swellings for Dup15q and Sei^TS1^ with pan-glial expression of constitutively active Fray^T206E^. n≥15. Statistical significance was determined by one-way ANOVA with Šídák’s multiple comparisons; ****, p<0.0001. FrayT206E overexpression suppressed nerve swellings compared to seizure mutants expressing control transgene, UAS-RFP.(I) Schematic model of SIK3 and Wnk converging on Fray to regulate glial K^+^ buffering. SIK3 phosphorylates HDAC4, sequestering it in the cytoplasm, allowing Mef2 to promote transcription of Aquaporin and Fray. Wnk phosphorylates Fray which promotes K+ buffering by activating NKCC and inactivating KCC.

Fray is a transcriptional target of the SIK3-HDAC4-Mef2 signaling cascade and a direct target of Wnk. Overexpression of constitutively active Fray is sufficient to suppress nerve swellings induced by loss of SIK3 and Wnk [13,25,26] (Fig 3A–3D). This led us to hypothesize that Fray may be a central node in the glial K^+^ buffering program. If so, then bypassing both the transcriptional and posttranslational programs via transgenic expression of a constitutively active Fray (Fray^T206E^) in glia might be sufficient to suppress seizure phenotypes in multiple hyperexcitable mutants. To test this, we expressed Fray^T206E^ in glia under the control of the pan-glia driver *Repo-Gal4* in each of the mutants. To assay seizure behavior, flies were subjected to seizure-inducing stimuli (42°C and 38°C water bath for temperature-sensitive Sei^TS1^ and NCKX^zyd^ mutants and a ten-second vortex for the bang-sensitive Dup15q model flies) and the percentage of animals seizing was recorded every ten seconds until all flies recovered (Fig 4A–4C). The cumulative area under the seizure curve was quantified and reported as an integrated seizure response. When constitutively active Fray was expressed in glia, seizure behavior was strongly suppressed in all three mutants (Fig 4D). As a second measure of seizure behavior that focuses on seizure initiation, we measured when the temperature sensitive mutants began to seize after exposure to elevated temperature (Fig 4E and 4F). This assay is only possible for the temperature sensitive mutants, as it is not feasible to assess initiation of seizures while the animals are being vortexed. To quantify seizure initiation, we calculated the time at which 50% of flies exhibit heat-induced seizures (Fig 4G). We used the pan-glial driver, Repo-Gal4, to drive the expression of constitutively active Fray (Fray^T206E^) in both Sei^TS1^ and NCKX^zyd^ mutants. This manipulation significantly delayed seizure initiation in each mutant.

Having demonstrated that constitutively active Fray potently blocks seizure behavior, we next assessed whether expression of constitutively active Fray was also sufficient to suppress the nerve swellings in the Sei^TS1^ and Dup15q mutants. To test this, we used the pan-glia driver, Repo-Gal4 to express Fray^T206E^ in both seizure mutants. The number of nerve swellings were significantly reduced when Fray^T206E^ was overexpressed, compared to expression of a control UAS-RFP. Taken together with the results above, we conclude that glial expression of activated Fray is sufficient to potently suppress multiple phenotypes of mechanistically distinct seizure mutants, highlighting the functional importance of Fray as an intersection point of the SIK3-dependent transcriptional program and Wnk-dependent posttranslational program for promoting ion homeostasis (Fig 4I).

### SIK3-HDAC4 signaling in cortex glia is important for seizure behavior

**Fig 5:**
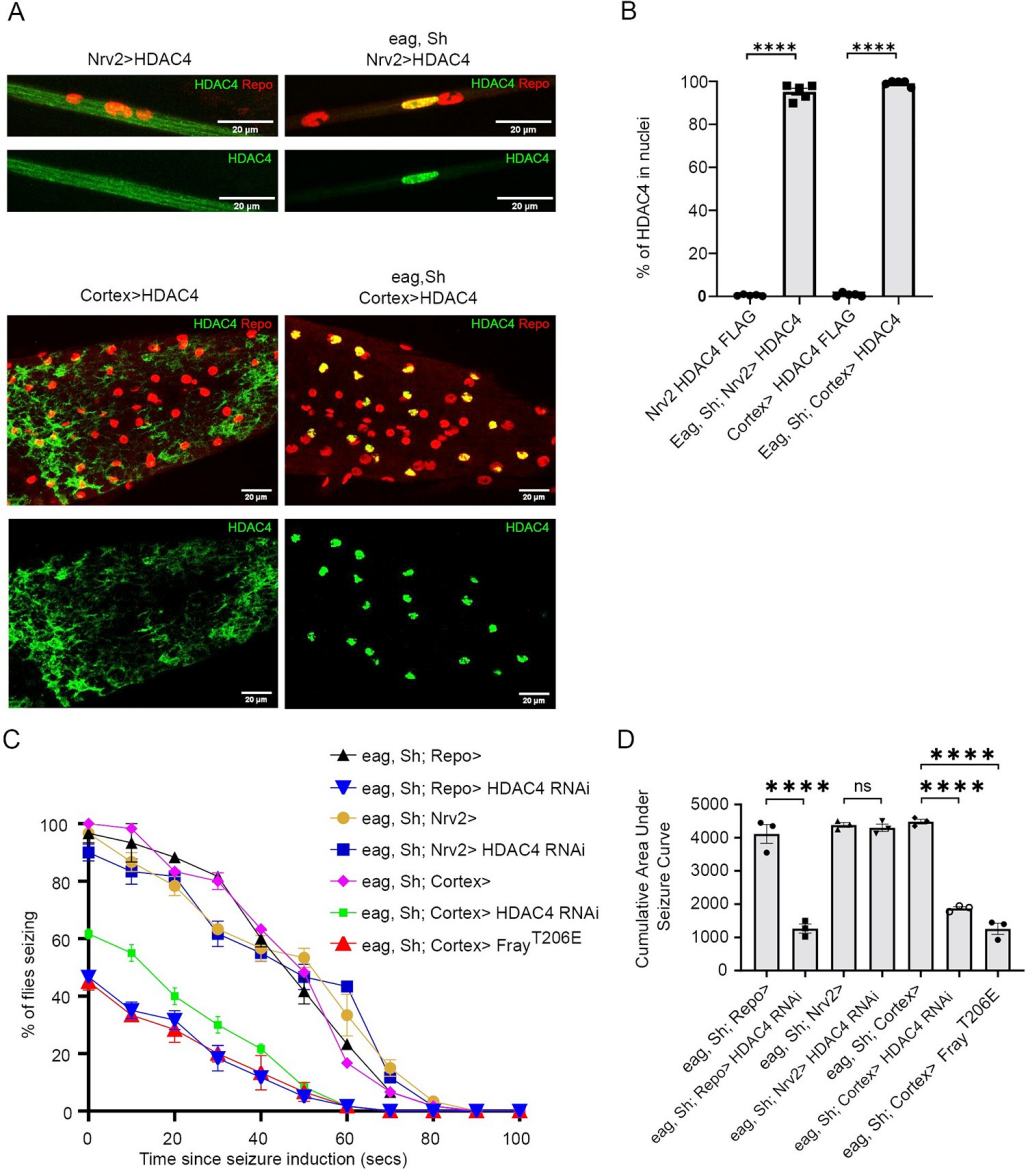
SIK3-HDAC4 signaling in Cortex glia is critical for seizure behavior. (A) Representative images of larval peripheral nerves showing HDAC localization in wrapping glia (*eag Shaker;* Nrv2>HDAC4) and cortex glia (*eag Shaker;* Cortex>HDAC4) of *eag Shaker* mutants. *eag Shaker* mutants induce nuclear shuttling of HDAC4 in both wrapping and cortex glia. (B) Quantification of glial nuclear HDAC4. Data are represented as the amount of HDAC4 in the nucleus divided by the total amount of HDAC4, expressed as a percentage (C) Time course of vortex-induced seizures in *eag Shaker* with HDAC4 knockdown in all glia (*eag Shaker;* Repo>HDAC4 RNAi), wrapping glia (*eag Shaker;* Nrv2> HDAC4 RNAi), and cortex glia (*eag Shaker;* Cortex> HDAC4 RNAi). UAS-LexA RNAi was used as a control transgene in each glial subtype (*eag Shaker; Repo>*, *eag Shaker;* Nrv2>, & *eag Shaker;* Cortex>). After seizure induction, the number of flies seizing were recorded every 10 seconds and plotted as a percentage of seizing flies. n≥ 3 groups of 10–20 flies per genotype per time point. (D) Quantification of area under the seizure curve for genotypes in (B). Statistical significance was determined using one-way ANOVA with Sidak’s multiple comparisons test; NS = not significant, **** p <0.0001.

We have shown that boosting K^+^ buffering in all glial cells is sufficient to suppress seizure behavior in *eag*, *Shaker* and other seizure mutants. Now, we seek to identify a specific glial subtype in which boosting buffering is sufficient to suppress seizure behavior. In our previous work, we demonstrated that SIK3-HDAC4 K^+^ buffering pathway is particularly critical in wrapping glia for suppressing extracellular edema in peripheral nerves [13]. However, seizures start in the central nervous system, so we wished to compare wrapping glia where we know the SIK3-HDAC4 pathway functions with CNS glial, where we predict K^+^ buffering is particularly important for seizure suppression. We focused on cortex glia because they wrap neuronal cell bodies in the central nervous system and participate in K^+^ buffering [15,17,18,27]. First, we assessed the state of the SIK3-HDAC4 signaling pathway in both wrapping glia in peripheral nerves and cortex glia in the CNS in the *eag*, *Shaker* mutant. We expressed FLAG-tagged HDAC4 exclusively in wrapping glia or cortex glia of wild type and *eag*, *Shaker* mutant flies via expression from selective Gal4 lines. We observed a dramatic re-localization of HDAC4 in both cell types from a diffuse cytosolic pattern in wild type flies to pronounced nuclear HDAC4 localization in the *eag Shaker* mutant (Fig 5A). Hence, the SIK3-HDAC4 K^+^ buffering pathway is inhibited in both wrapping glia and cortex glia in this hyperexcitable mutant.

Next, we asked whether the inhibition of the SIK3-HDAC4 K^+^ buffering pathway in either glial subtype contributes to seizure behavior by testing whether enhancing K^+^ buffering in cortex glia could suppress seizure behavior in *eag Shaker*. We enhanced K^+^ buffering, using wrapping glia and cortex glia specific Gal4 drivers to knock down HDAC4. In wrapping glia, HDAC4 knockdown did not influence seizure behavior (Fig 5B and 5C), even though we previously demonstrated that this is the key cell type for controlling peripheral edema [13]. In contrast, in cortex glia, HDAC4 knockdown dramatically suppressed seizure behavior, leading to a significant decrease in the integrated seizure response compared to *eag Shaker* flies expressing control transgenes (Fig 5B and 5C). If cortex glia is a critical glial subtype for seizure behavior, we would expect overexpression of constitutively active Fray to also suppress seizure behavior. When constitutively active Fray was expressed in cortex glia of *eag Shaker* mutants, we observed dramatic seizure suppression as indicated by the significant decrease in integrated seizure response. Hence, boosting K^+^ buffering in cortex glia is sufficient to suppress seizure behavior, highlighting the importance of cortex glia for the regulation of neuronal excitability. In conjunction with our prior work, these findings demonstrate that K^+^ buffering in different glia subtypes is necessary for distinct physiological functions.

### Enhancing K+ buffering in cortex glia of NCKX^zyd^ is sufficient to suppress seizure behavior

**Fig 6:**
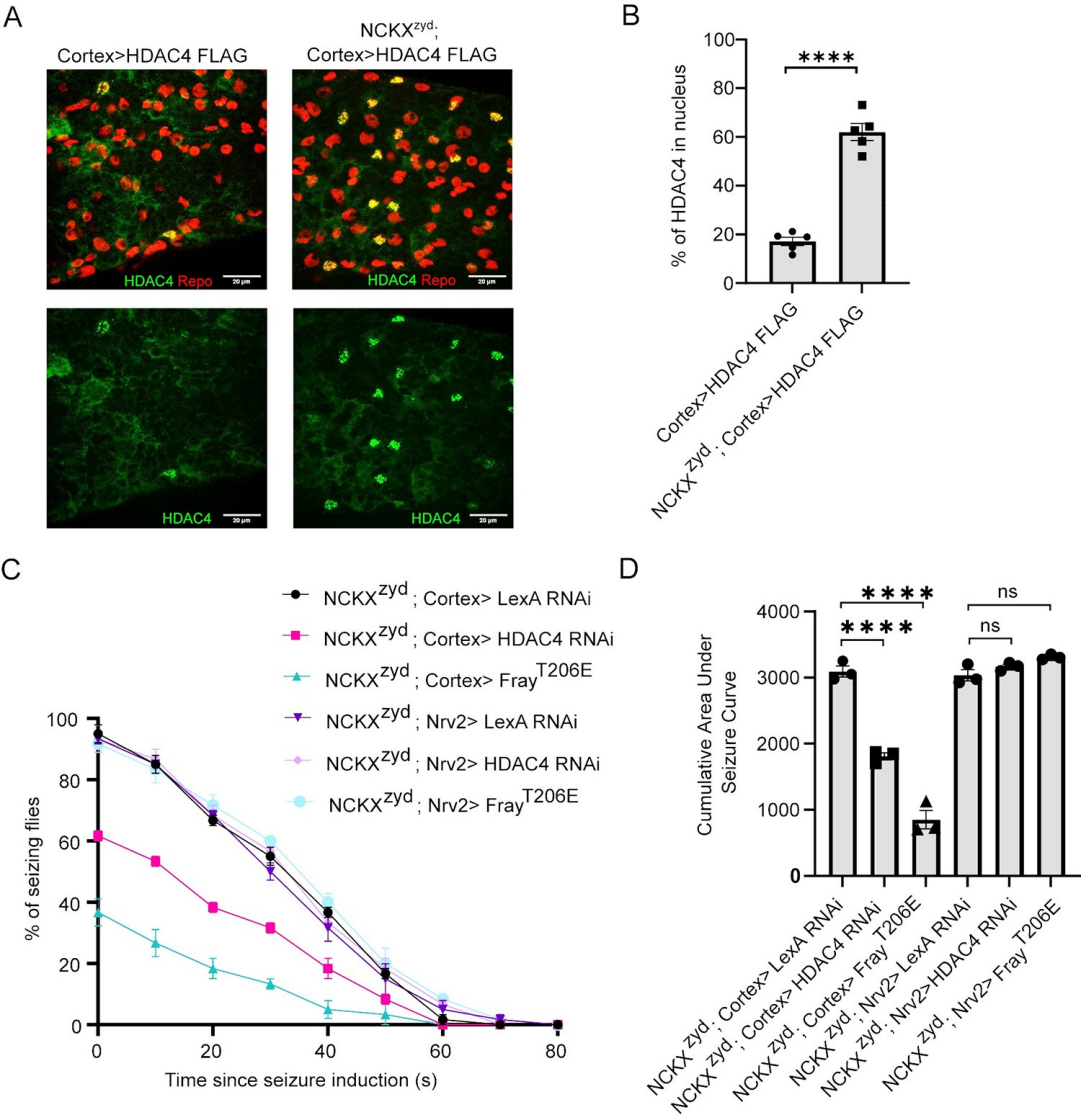
Enhancing glial K^+^ buffering in cortex glia suppresses seizure behavior in NCKX^zyd^. Representative images of ventral nerve cord showing HDAC4 localization in cortex glia of NCXK^zyd^ mutants. (B) Quantification of percentage of HDAC4 in glial nuclei for genotypes in (A). Data are represented as HDAC4 in the nucleus divided by the total amount of HDAC4, expressed as a percentage. (C) Time course of vortex-induced seizures in NCKXzyd with Fray overexpression or HDAC4 knockdown in wrapping glia (NCKXzyd; Nrv2>) and cortex glia (NCKXzyd; Cortex glia). UAS-LexA RNAi was used as a control transgene in each glial subtype. After seizure induction, the number of flies seizing were recorded every 10 seconds and plotted as a percentage of seizing flies. n≥3 groups of 10–20 flies per genotype per time point. (D) Quantification of area under the seizure curve for genotypes in (B). Statistical significance was determined using one-way ANOVA with Sidak’s multiple comparisons test; NS = not significant, **** p <0.0001.

The NCKX^zyd^ mutant induces seizures by disupting Ca^2+^ signaling and regulation of endocytosis of the sandman K^+^ channel in cortex glia [18]. Above, we showed that the SIK3/Fray pathway also functions in cortex glia in the *eag Shaker* mutant. To investigate whether the NCKX^zyd^ mutant also impacts the SIK3/Fray pathway in these cells, we used cortex glia-specific Gal4 drivers to express FLAG-tagged HDAC4 in wild type and NCKX^zyd^ flies. We observed a clear re-localization of HDAC4 from a primarily diffuse cytosolic pattern in wild type flies to a pronounced nuclear localization in NCKX^zyd^ mutants (Fig 6A and 6B), suggesting that the SIK3-HDAC4 K^+^ buffering pathway is inhibited in cortex glia of this hyperexcitability mutant. This is in contrast to wrapping glia where the NCKX^zyd^ mutant did not influence HDAC4 localization (Fig 1). Next, we asked whether the inhibition of SIK3-HDAC4 K^+^ buffering pathway in cortex glia contributes to the seizure behavior of the NCKX^zyd^ mutants by testing whether reactivating this pathway in cortex glia could suppress their seizure behavior. To enhance K^+^ buffering in cortex glia, we used a cortex specific Gal4 driver to knockdown HDAC4 or overexpress a constitutively active Fray in wild type and NCKX^zyd^ mutant flies. Enhancing K^+^ buffering in cortex glia dramatically suppressed seizure behaviors in NCKX^zyd^ mutants, leading to a significantly decreased integrated seizure response compared to NCKX^zyd^ mutants expressing a control transgene (Fig 6C and 6D). Enhancing K^+^ buffering in wrapping glia did not influence seizure behavior, highlighting the importance of cortex glial regulation of seizure behavior. These findings show that the NCKX^zyd^ mutant impacts not only Ca^2+^ signaling and sandman endocytosis [18] but also the SIK3 K^+^ buffering pathway, and that both pathways influence seizure behavior, demonstrating that these two K^+^ buffering pathways are not strictly independent.

## Discussion

Here we demonstrate that enhancing glial ionic buffering is sufficient to suppress seizure behavior across a variety of *Drosophila* hyperexcitability models. We show that *Fray*, an important upstream regulator of glial K^+^ transporters [11] and a transcriptional target of SIK3-HDAC4 signaling [13], is also a substrate for the kinase Wnk in glia, and that Wnk-dependent regulation of Fray is required for proper ionic buffering in glia. Overexpressing a constitutively active Fray, bypassing both SIK3- and Wnk-dependent regulation, potently suppressed seizure behavior in molecularly distinct hyperexcitability mutants. These findings suggest that Fray is a central convergence point between two major glial ion homeostasis regulatory pathways. Finally, we identify cortex glia as central for the control of neuronal excitability, as enhancing K^+^ buffering specifically in this glial subtype is sufficient to suppress seizure behavior across diverse hyperexcitability mutants. Taken together, these findings emphasize the centrality of glial ionic buffering in the control of neuronal excitability, deepen our understanding of the molecular mechanisms controlling glial ionic homeostasis, and highlight the potential therapeutic utility of bolstering glial K^+^ buffering as a new paradigm for the treatment of seizure disorders.

### Promoting glial K^+^ buffering suppresses seizure behavior

In our previous study, we made the surprising observation that the SIK3 glial ionic buffering program is inactivated in *eag Shaker*, a classic seizure mutant [14]. We provided evidence that the program is turned off by glia for self-protection, as re-activating this program induces glial cell swelling and activates JNK, a central cell stress signaling pathway. However, turning off the glial SIK3 program comes at the cost of exacerbating neuronal hyperexcitability. Indeed, reactivation of the SIK3 glial ionic buffering program is sufficient to suppress seizure behavior and dramatically extend lifespan in the *eag Shaker* mutant [14]. Here we investigated whether this glioprotective inactivation of K^+^ buffering was a peculiarity of the *eag Shaker* mutant, or a more general feature of hyperexcitability mutants. We found evidence that the pathway is turned off in two additional mutants, a model of human *Dup15q* syndrome in which the ubiquitin ligase Ube3A is overexpressed in glia [21], and the NCKX^zyd^, in which a glial K^+^ -dependent Na+/Ca2+ exchanger is mutant ([17,18]). In addition, we found that all tested mutants could be suppressed by activation of the SIK3 K^+^ buffering program in glia. We and others previously demonstrated that a decrease in glial K^+^ buffering can promote seizure behavior [13, 18,21,23]. Our current findings indicate that hyperexcitable mutants may often inhibit glial K^+^ buffering, and that reactivation of the glial K^+^ buffering program may be a general mechanism to inhibit seizure behavior and so could have potential as a treatment for seizure disorders.

### Fray is a convergence node for distinct ion homeostasis regulatory mechanisms

Fray was the first protein demonstrated to be required for glial ion homeostasis in *Drosophila* [11,12], where it phosphorylates and activates the cation-chloride transporter Ncc69 to promote proper ion and water buffering in peripheral nerve [12]. In addition, we demonstrated that Fray is one of the downstream genes activated by the SIK3-HDAC4-Mef2 glial transcriptional pathway. Here we demonstrate that Wnk is also required for proper ion and water homeostasis in *Drosophila* glia, and that Wnk functions through Fray (Fig 3). Hence, both the transcriptional SIK3 pathway and post-translational Wnk pathway converge on Fray to regulate ion homeostasis. Moreover, we showed via transgenic overexpression of a constitutively active Fray that this single protein, when active, is sufficient to promote glial ion and water homeostasis and suppress neuronal excitability in a variety of seizure mutants (Fig 4). The identification of Fray as a convergence node for these two pathways has interesting implications for regulatory mechanisms controlling the balance between glial uptake of ions, which can be neuroprotective but lead to glial swelling and damage, and glial cell volume regulatory mechanisms that preserve glial health at the expense of extracellular ion accumulation and increased neuronal excitability. We previously showed that with hyperexcitability the monoamine octopamine signaled to glia to turn down the SIK3 transcriptional program and, presumably, decrease the expression of Fray [14]. While such a mechanism would be a powerful approach to reduce glial ion uptake, it is also expected to be quite slow. With the identification of Wnk as a required activator of Fray, there is now the likelihood of a second, faster mechanism to inhibit glial ion uptake. Wnk is a potassium-sensitive kinase—high levels of intracellular K^+^ inhibit Wnk activity [28], providing a direct mechanism for excess K^+^ uptake to rapidly blunt glial ion uptake and protect the glia from osmotic stress. Future studies will address whether hyperexcitability mutants inhibit Wnk activity in glia and whether there may be additional cross talk between the SIK3 and Wnk pathways.

### Cortex glia is a critical glial subtype for seizure susceptibility

Much like their mammalian counterparts, *Drosophila* glial cells have specialized functions including phagocytosis, maintenance of the blood-brain barrier, neurotransmitter reuptake, and ion homeostasis [15,29,30]. In our previous work, we demonstrated that SIK3-HDAC4 K^+^ buffering pathway was particularly critical in wrapping glia for suppressing extracellular edema in peripheral nerves, and that the SIK3-HDAC4 K^+^ buffering program was turned off in peripheral glia in the *eag*, *Shaker* mutant [13,14]. However, we show here that enhancing the K^+^ buffering program in wrapping glia failed to suppress seizures in the *eag*, *Shaker* mutant (Fig 5). We reasoned that seizures start in the central nervous system and propagate to the periphery, and so there must be a different glial subtype in the central nervous system contributing to seizure behavior. We focused on cortex glia, which wrap neuronal cell bodies and can participate in K^+^ buffering [18], and which we found also turn off the SIK3 K^+^ buffering program in *eag*, *Shaker*. As we predicted, activation of the glial ion buffering program exclusively in cortex glia robustly suppressed seizures in this mutant (Fig 5). Moreover, these findings are not specific for *eag*, *Shaker*, as the NCKX^zyd^ mutant also inhibits the SIK3-HDAC4 K^+^ buffering pathway in cortex glia, and reactivation of this program in these cells again dramatically suppresses seizure behavior. Cortex glia are likely important for regulating excitability because they control the extracellular ionic milieu around the site of action potential initiation. Taken together these finding indicate that multiple glial subtypes contribute to ionic buffering around distinct portions of the neuron, and our studies do not exclude the possibility that additional glial subtypes may also contribute to ion homeostasis. Nonetheless, we suggest that methods to selectively target mammalian astrocytes at the axon initial segment could be a powerful approach for novel therapies to inhibit neuronal hyperexcitability.

## Materials and methods

### Fly stocks

Fly stocks were maintained using standard techniques, cultured at 25°C in 60% relative humidity. The following stocks were used in this study: Repo-Gal4 (BDSC #7415); R54H02-Gal4 (BDSC #45784); Nrv2-Gal4 (BDSC# 6799); UAS-Ube3A (BDSC# 90376); UAS-Wnk RNAi (BDSC #62150; #42521); UAS-Fray RNAi (BDSC #55878; #42569); UAS-HDAC4 RNAi (BDSC #28549; #34774); UAS-LexA RNAi (BDSC #67945); UAS-mCD8-RFP (BDSC# 32218); *Sei*^*TS1*^, *Eag*^1^ *Shaker*^120^ and *NCKX*^*zydeco*^ are gifts from Troy Littleton; UAS-RnSPAK^D219A^, UAS-Wnk^WT^, and UAS-Fray^T206E^ were gifts from Aylin Rodan.

### Immunocytochemistry

Third instar larvae were dissected in ice-cold phosphate-buffered saline (PBS) and immediately fixed with 4% paraformaldehyde for 20 minutes at room temperature. After fixing, larvae were washed with PBS + 0.1% Triton X-100 (PBS-T) 3 times (3X) for 10 minutes, followed by blocking in PBS-T +5% goat serum. All antibodies were incubated with 5% goat serum. To examine peripheral nerve morphology, larvae were incubated in Cy3-conjugated goat α-HRP antibody (1:1000; Jackson ImmunoResearch cat # 123-165-021, RRID: AB 2338959) for 60 minutes at room temperature. In all other experiments, larvae were incubated overnight at 4°C in the following primary antibodies: rabbit α-FLAG (1:1000; Cell Signaling, cat# 147935); mouse α-Repo (1:100; Developmental Studies Hybridoma Bank, RRID_AB528448). After overnight incubation, primary antibodies were washed 3X with PBS-T for 10 minutes at room temperature. Larvae were then incubated with the following secondary antibodies for 90 minutes; AlexaFluor 488-conjugated goat α-rabbit (1:1000; Invitorgen, cat# A-10520, RRID:AB_2534029); Cy3 goat α-mouse (1:1000; Jackson ImmunoResearch, cat# A-10520: AB_2534029); 647 AffiniPure goat α-HRP (1:1000; Jackson ImmunoResearch, cat# A-123-065-021). After three, 10-minute wash in PBST, larvae were transferred to be equilibrated in 70% glycerol in PBS for at least 1 hour. Larvae were then mounted in Vectashield (Vector Laboratories) and ready to be imaged.

### Western Blots (Measurement of Drosophila WNK activity)

20 flies brains expressing a kinase-dead rat SPAK^D219A^ in glial cells were homogenized in RIPA buffer with protease inhibitor (Millipore Sigma cat# 11873580001) and phosphatase inhibitor (Sigma-Aldrich, cat# p0044) to create protein sample. The protein samples were spun down at max speed (10–11,000g) for 15 minutes at 4°C. Lysates were created from the supernatant. Protein concentration was determined by BCA Protein Assay Kit (Thermo Scientific; cat# 23225).Samples were run on 4–20% Mini-PROTEAN TGX Precast Protein Gels (Bio-Rad Laboratories, cat# 4561093). Membranes were washed with Tris-buffered saline with 0.2% Tween-20 (TBS-T) followed by a 1-hour block of a TBS-T + 5% milk. Gels were transferred to nitrocellulose membranes using Trans-Blot Turbo Midi Nitrocellulose Transfer Packs (Bio-Rad Laboratories, cat# 1704159). Membranes were blocked in 5% bovine serum albumin (Sigma-Aldrich, cat# A3912-50G) in TBS + 0.1% tween for 60 min and incubated overnight at 4°C with the following primary antibodies: rabbit anti-pSPAK (1:2500; Millipore No. 07–2273); rabbit anti-tSPAK (1:1000; Cell Signaling Technology #2281); rabbit anti β-actin (1:1000; Cell Signaling Technology cat# 4967S). On the following day, primary antibodies were washed 3x with TBS-T for 10 minutes. Membranes were then incubated in the following secondary antibodies in TBS-T + 5% milk for 60 minutes at room temperature: Peroxidase AffiniPure Goat anti-Rabbit IgG (1:5,000;Jackson ImmunoResearch Laboratories cat# 111-035-045). Blots were stripped using Restore PLUS Western Blot Stripping Buffer (Thermo Fisher Scientific cat# 46430) for 15 minutes at 37°C before being re-probed for total SPAK. Western blots were developed with Immobilon Western Chemiluminescent Substrate (EMD Millipore) and imaged on a G:Box Chemi-XX6 (Syngene). Blots were quantified using ImageJ (NIH, v1.53c). First, phosphorylated-SPAK (pSPAK) and total SPAK (t-SPAK) were first normalized to their respective loading control (β-Actin) and then phosphorylated protein was divided by total protein such that the result was (p-SPAK/β-Actin)/ (t-SPAK/β-actin). Phosphorylation was calculated by measuring the ratio of p-SPAK/t-SPAK. The Repo>RnSPAK^D219A^ condition demonstrates how much SPAK was phosphorylated at baseline, so we generated average intensity for this condition, and then normalized all the datapoints by dividing by this average, thereby setting the value of the Repo>RnSPAK^D219A^ condition to 1. Then we compared Repo>Wnk RNAi and Repo>Wnk^WT^ to Repo> RnSPAK^D219A^.

### Seizure behavior assay

Adult flies were collected within 24 hours post-eclosion and rested for 4 hours before being assayed for seizure behavior. Flies were transferred to an empty culture vial in groups of 10–20 and subjected to seizure-inducing stimuli. For bang-sensitive mutants (*Eag*^*1*^ *Shaker*^*120*^*)* flies were transferred to an empty vial in groups of 10–20 and vortexed for 15 seconds at max speed using a vortex mixer (Fisher Scientific). For temperature-sensitive mutants flies were transferred to preheated vials and submerged in a 38°C (NCKX^zyd^) water bath or 42°C water bath (Sei^TS1^) for 1–2 minutes. After seizure induction, the number of seizing flies were recorded every 10 seconds until all flies recovered. Seizure behaviors included shuddering, leg shaking, spinning flight and contorted posture. A minimum of 50 flies were tested for each genotype and condition.

### Seizure susceptibility test

Adult flies were collected within 24 hours post-eclosion and rested for 4 hours before being assayed for seizure behavior. Flies were transferred to preheated vials and submerged in a 38°C (NCKX^zyd^) water bath or 42°C water bath (Sei^TS1^). Following submersion, the number of seizing flies (paralysis, shuddering, leg shaking, spinning flight and contorted posture) were recorded every 10 seconds. The time at which it took 50% of the flies to exhibit seizure behavior was used as a measure of seizure susceptibility. A minimum of 40 flies were tested for each genotype and condition.

### Imaging and analysis

For peripheral nerve morphology, larvae peripheral nerves were stained for α-HRP and imaged using 20/0.60 NA and 40/1.15 NAoil immersion objectives on a Leica TCS SPE confocal microscope, a Leica DFC7000 T camera, andthe LAS X software. We acquired images for all genotypes and conditions in the same experiment inone sitting using identical laser power, gain, and offset settings. Images shown are maximal Z-projections of confocal stacks, except for thin optical sections used to highlight glial swellings. Photo-shop CC (2.2, Adobe) and Illustrator (15.0.0, Adobe) were used to minimally process the images in preparation for final figures. Nerve swellings were quantified by identifying and scoring regions with a maximum nerve width >15mm. The total number of nerve swellings was measured for each larvae and subsequently averaged for each genotype and condition.

For HDAC4 localization in peripheral nerves, larvae were stained for FLAG-tagged HDAC4 in glia and repo-labeled glial nuclei. Glial nuclei were stained with mouse α-Repo antibody, HDAC4 was labeled with rabbit α-FLAG, and nerves were labeled with 647a-HRP antibody. Their peripheral nerves were imaged using 40/1.15 NA oil immersion objective on the confocal microscope and other equipment mentioned above. Images were acquired under identical settings and shown as maximal Z-projections of confocal stacks. Fluorescence intensity of FLAG-tagged HDAC4 was analyzed using Image J (1.52 k, National Institute of Health). The method used to quantify HDAC4 localization in glial nuclei and cytosol was described in a previous study. In brief, we used the red channel (Repo) to generate a nuclear mask and applied this mask to the FLAG channel to selectively measure HDAC4 levels in glial nuclei. To assess HDAC4 levels in glial cytoplasm, FLAG signals in the ‘non-nuclei’ areas were also analyzed by subtracting the mask from total area of the peripheral nerve. The nuclear:cytoplasmic ratio of HDAC4 intensity was then calculated using these two measurements. A minimum of 4–5 independent peripheral nerves per larvae was examined for each genotype and condition and normalized to the control. The SEM of each larvae was plotted as an individual point. At least 6 larvae were used for each genotype and condition. For HDAC4 localization in cortex glia, the amount of HDAC4 in the nucleus was divided by the total amount of HDAC4 in the ventral nerve cord. This was multiplied by 100 and reported as percentage of HDAC4 in the nucleus.

### Experimental design and statistical analysis

For immunocytochemistry, a minimum of 15 larvae were assessed for each genotype and condition. For behavioral studies, a minimum of 40 flies were assessed per genotype. Male and female flies were used in comparable numbers except for seizure mutants on the X-chromosome (*NCKX*^*zyd*^ & *eag*^*1*^ *Shaker*^*120*^), in which males were used. There are no statistical differences between the two groups.

All data are shown as mean ± SEM. Data has passed the ‘Agostino-Pearson and the Shapiro-Wilk test for normality before being evaluated for statistical significance. Statistical analyses were performed using Prism (7.02, GraphPad), including one-way ANOVA test with Dunnett’s and Šídák’s multiple comparisons test. Results shown represent data pooled from at least 3 independent experiments performed on larvae or adult flies derived from different crosses. Researchers were blinded to genotype and treatment condition during all experiments and data analyses.

## Supporting information

S1 Data Excel graph with the data that underlie graphs throughout the manuscript. (XLSX)
